# Design and feasibility of a novel program of cervical screening in Nigeria: self-sampled HPV testing paired with visual triage

**DOI:** 10.1186/s13027-020-00324-5

**Published:** 2020-10-14

**Authors:** Kanan T. Desai, Kayode O. Ajenifuja, Adekunbiola Banjo, Clement A. Adepiti, Akiva Novetsky, Cathy Sebag, Mark H. Einstein, Temitope Oyinloye, Tamara R. Litwin, Matt Horning, Fatai Olatunde Olanrewaju, Mufutau Muphy Oripelaye, Esther Afolabi, Oluwole O. Odujoko, Philip E. Castle, Sameer Antani, Ben Wilson, Liming Hu, Courosh Mehanian, Maria Demarco, Julia C. Gage, Zhiyun Xue, Leonard R. Long, Li Cheung, Didem Egemen, Nicolas Wentzensen, Mark Schiffman

**Affiliations:** 1 Division of Cancer Epidemiology and Genetics, National Cancer Institute, NIH, Rockville, USA; 2 Oak Ridge Institute of Science and Education, Oak Ridge, USA; 3 Department of Obstetrics and Gynecology, Obafemi Awolowo University Teaching Hospital, Ile Ife, Nigeria; 4 Lagos University Teaching Hospital, Lagos, Nigeria; 5 Rutgers New Jersey Medical School and Cancer Institute of New Jersey (CINJ), Newark, USA; 6 Hela Health, Tel Aviv, Israel; 7 Global Health Labs, Bellevue, USA; 8 Albert Einstein College of Medicine, New York City, USA; 9 National Library of Medicine, NIH, Bethesda, USA

**Keywords:** Cervical screening, HPV, Self-sampling, Triage, Automated visual evaluation

## Abstract

**Background:**

Accelerated global control of cervical cancer would require primary prevention with human papillomavirus (HPV) vaccination in addition to novel screening program strategies that are simple, inexpensive, and effective. We present the feasibility and outcome of a community-based HPV self-sampled screening program.

**Methods:**

In Ile Ife, Nigeria, 9406 women aged 30–49 years collected vaginal self-samples, which were tested for HPV in the local study laboratory using Hybrid Capture-2 (HC2) (Qiagen). HPV-positive women were referred to the colposcopy clinic. Gynecologist colposcopic impression dictated immediate management; biopsies were taken when definite acetowhitening was present to produce a histopathologic reference standard of precancer (and to determine final clinical management). Retrospective linkage to the medical records identified 442 of 9406 women living with HIV (WLWH).

**Results:**

With self-sampling, it was possible to screen more than 100 women per day per clinic. Following an audio-visual presentation and in-person instructions, overall acceptability of self-sampling was very high (81.2% women preferring self-sampling over clinician collection). HPV positivity was found in 17.3% of women. Intensive follow-up contributed to 85.9% attendance at the colposcopy clinic. Of those referred, 8.2% were initially treated with thermal ablation and 5.6% with large loop excision of transformation zone (LLETZ). Full visibility of the squamocolumnar junction, necessary for optimal visual triage and ablation, declined from 68.5% at age 30 to 35.4% at age 49. CIN2+ and CIN3+ (CIN- Cervical intraepithelial neoplasia), including five cancers, were identified by histology in 5.9 and 3.2% of the HPV-positive women, respectively (0.9 and 0.5% of the total screening population), leading to additional treatment as indicated. The prevalences of HPV infection and CIN2+ were substantially higher (40.5 and 2.5%, respectively) among WLWH. Colposcopic impression led to over- and under-treatment compared to the histopathology reference standard.

**Conclusion:**

A cervical cancer screening program using self-sampled HPV testing, with colposcopic immediate management of women positive for HPV, proved feasible in Nigeria. Based on the collected specimens and images, we are now evaluating the use of a combination of partial HPV typing and automated visual evaluation (AVE) of cervical images to improve the accuracy of the screening program.

## Introduction

Nearly 85% of the annual 570,000 cervical cancer cases and almost 90% of the 311,000 related deaths occur in lower-resource countries [1, 2] due to the lack of effective cervical cancer prevention programs [3]. The COVID-19 (coronavirus disease of 2019) pandemic threatens to reduce elective procedures even further, including cervical screening and related diagnostic procedures, especially in lower-resource settings, and will likely worsen cervical cancer health disparities. As a major advantage compared with cytology, the specimen for HPV (human papillomavirus) testing can be collected by the woman herself using a vaginal self-collection device, yielding sensitivity for HPV infection that is similar to clinician-collected specimens when target-amplification methods like PCR (polymerase chain reaction) are used [4, 5]. Use of HPV testing will require a second diagnostic modality for positives, because majority of HPV infections are cleared within 1–2 years of initial detection. Only HPV infections that persist can cause precancer and invasive cancer [6]. Thus, an improved screening program must include triage methods to focus treatment safely on the small fraction of HPV-positive women with precancer, the general term we use to refer to lesions at substantial risk of invasion without treatment [7]. Ideal characteristics of a triage test for HPV-positive women, for use in low-resource regions, would include excellent risk discrimination (high precancer risk in positives, low risk in negatives), low-cost, simplicity, and point-of-care use.

A new candidate for triage of HPV-positive women is automated visual evaluation (AVE) of cervical images using a deep-learning algorithm. A proof-of-principle evaluation of AVE of Cervigrams (NTL Worldwide, Fenton, Missouri) for the diagnosis of cervical precancer, demonstrated higher accuracy of AVE than expert gynecologist visual assessment or cytology [8].

Although these are promising results, the Cervigram cervical images were based on film and a discontinued expensive custom camera, and the technology is obsolete. Thus, it is essential to advance the transfer of the method to modern image acquisition devices (e.g., smartphones) [9]. There is also a need to evaluate the performance of AVE specifically for the triage of HPV-positive women, as HPV-positive controls [<CIN2 (CIN-cervical intraepithelial neoplasia)] tend to look less normal and are consequently harder to differentiate from precancer cases than HPV-negative controls.

This paper describes the field methods, feasibility, and preliminary descriptive results of Project Itoju in Ile-Ife, Nigeria, designed to evaluate ultimately the combined strategy of self-sampled HPV typing combined with AVE triage on three different image capture devices.

## Materials and methods

### Study design and overview

Women aged 30–49 years (comprising ~ 8% of the total population [10]) residing in the catchment area of Obafemi Awolowo University Teaching Hospitals Complex (OAUTHC) in Ile-Ife, Nigeria were invited by a public message campaign for self-sampled HPV testing. Women who screened HPV-positive were referred to the colposcopy clinic and invited to participate in a research study (examining triage methods) under informed consent. Standard colposcopic examination, including colposcopic images and treatment, if indicated, was offered to all HPV-positive women regardless of participation in the triage methods research. In addition, cervical images were collected with a cellphone and the EVA (enhanced visual assessment) system (MobileODT, Israel), and a cervical sample was collected for subsequent HPV typing, from all the participants in the study.

The study was approved by National Cancer Institute (NCI) and OAUTHC ethical Institutional Review Boards.

### Study timeline

The screening period was from November 2018 to December 2019. The colposcopy period was from December 2018 to March 2020.

### Screening visit

The screening visit started when a woman attended one of the three screening clinics (no appointment needed). A nurse-administered short screening questionnaire determined eligibility [age 30–49 years and not pregnant (self-reported)] for screening. If a woman was menstruating and was uncomfortable undergoing screening, she was advised to return later. Known pregnant women were excluded due to an “abundance of caution”, mainly to prevent any possibility that an unrelated adverse pregnancy outcome could be due, or even perceived to be due, to the self-sampling for screening. The lower limit of age for screening was set at 30 years since younger women have a high prevalence of HPV but a very low risk of cancer [11]. The upper age limit was restricted to 49 years due to age-related repositioning of the squamocolumnar junction (SCJ), where cancers arise, into the endocervical canal, limiting the ability of any existing visual screening or triage method to diagnose precancer accurately [12]. An informed consent at screening was obtained from eligible women seeking permission to store the left-over sample after HPV testing for future research and to be contacted in future for a follow-up study. However, the screening effort was a public health intervention and not an experimental study.

After enrollment, eligible women were provided with an HPV self-sample collection kit containing a cervical brush and a Specimen Transport Medium (STM) tube (Qiagen, USA) [13]. Women were shown a 5-min animated video on how to collect a vaginal self-sample [https://www.youtube.com/watch?v=JiNqrDntbTc] [14] while waiting. Women went into a private self-sample collection area one at a time to self-sample. After collecting the sample, each woman left the brush in the STM vial in a rack. A nurse helped to break the stem of the brush, closed and labelled the vial, and cleaned the outside of the vial and rack with an alcohol wipe. The nurse was available to assist women in specimen collection upon request. The collection room was cleaned between collections. Before leaving the clinic, participants completed an anonymous feedback form regarding their experience with self-sampling. The specimens were stored at room temperature and transferred to the HPV laboratory at the end of the day, to be stored at 2–8^0^ C until testing.

### HPV testing

The primary HPV test used in the study was the Digene Hybrid Capture-2 (HC2) HPV DNA (deoxyribose nucleic acid) Test (Qiagen, USA), which is a US Food and Drug Administration approved nucleic acid hybridization assay with signal amplification using microplate chemiluminescence targeting 13 high-risk types of HPV DNA in cervical and vaginal specimens, without distinguishing between them [15].

Trained nurses called participants by phone, when their HPV test results were available (within two weeks of collection for most of the study period). HPV-negative women were informed and educated about the test result over the phone, and their questions were answered. HPV-positive women were asked to visit the colposcopy clinic to receive their test results. A minimum of five contact attempts were made to approach and advise a woman to attend the colposcopy clinic before a woman was declared lost to follow-up.

### Colposcopy clinic visit

At the clinic, a nurse communicated the positive HPV test result and its clinical meaning to the woman, ensuring privacy. The woman was counseled to undergo a colposcopy examination, preferably during that same visit or later.

Each woman was registered and interviewed to determine eligibility for the triage methods research study (analyses in progress to be reported separately). Anyone with a history of cervical cancer, hysterectomy, or who was pregnant at the time of enrollment (confirmed with rapid pregnancy test) was excluded from the study. A female nurse took informed consent from all the eligible women for participation in a research study (examining triage methods). An anonymized picture of the cervix was shown to the woman at the time of consent in order to reassure her about confidentiality and privacy of image collection and to minimize refusals.

Following the interview, a colposcopy examination was performed by one of the study gynecologists (KOA, CAA). Cervical images for research were collected one minute after applying 5% acetic acid for each device, sequentially with three different devices: 1) a Samsung Galaxy S8 [16] smartphone; 2) a MobileODT EVA device that provided lighting and magnification for a Samsung Galaxy J5 smartphone [17]; and 3) a Zeiss FC150 colposcopic image captured via a beam splitter by a DSLR (digital single-lens reflex) camera [18, 19]. After image collection, a cervical specimen was collected using a cervical sample collection kit containing a cervical brush and a STM tube (Qiagen, USA) [13] and stored at 2-8 °C. We plan to test this sample along with the residual sample from screening for HPV typing using the TypeSeq HPV test [20] at the NCI and report the results in future publications.

At the beginning of the study, a dry swab sample was collected at colposcopy, before collecting images and applying acetic acid, for the two-type OncoE6 test (Arbor Vita, USA). The test is known to have high positive predictive value; in fact, three of the five positives (all for HPV 16) were diagnosed with CIN2+. However, the test was dropped after testing 373 samples due to rare positivity and low yield.

Finally, a standard colposcopy examination was performed to assess the presence and possible nature of cervical lesions and to take biopsies of acetowhite lesions, up to a maximum of four biopsies. In addition, endocervical curettage (ECC) was performed in cases where the SCJ was not fully visible, even in the absence of acetowhitening. All women with acetowhite lesions were offered immediate treatment without waiting for histopathology results, following the American Society of Colposcopy and Cervical Pathology 2012 Consensus guidelines [21], leaning towards the more clinically-aggressive options for women at risk of being lost to follow-up. Either thermal ablation or large loop excision of the transformation zone (LLETZ) was performed, depending on the colposcopy examination findings. Ablation was performed only if the SCJ was fully visible, the entire lesion was visible, the lesion did not cover > 75% of the ectocervix, and cervix architecture was appropriate for the ablation probe [22].

### Histopathology and final diagnosis

Histopathological confirmation of CIN2 or CIN3 was used as the reference standard for the presence of precancer, against which other experimental tests were evaluated and final clinical review decisions were made. Even though from the clinical management purposes, all high-grade (CIN2+) lesions were treated equivalently; for the true yield of the screening effort, we reported the prevalence of CIN2+ and CIN3+ lesions separately to avoid the ambiguity of equivocal CIN2 lesions (a mixture of HPV infections, true precancers, and an error in histopathologic diagnosis) [23]. The study pathologist (AB) at the University of Lagos performed all pathology diagnoses.

### Quality assurance review and treatment recalls

All cases were reviewed for adequate clinical management by a US gynecologic oncologist (AN). The more complex cases were discussed in a case conference call. Recall was recommended for women needing further management and such cases were re-reviewed once the recall was completed until the case was determined to be adequately treated. We planned to reach all women needing recall for additional management, a minimum of seven times. However, we restricted our attempts, and recalled only those women at the highest immediate risk of invasive cancer because of the spread of the COVID-19 pandemic in March 2020.

### Screening and management project software

All data and images were collected with a HIPAA (Health Insurance Portability and Accountability Act) compliant smartphone application ‘EVA for research’ (MobileODT, Israel). The data platform was custom designed for the project (led by CS) using an advanced barcode scanning system to limit human error from manual key-in. The data and images from different sources on study assigned smartphones were held locally until internet connectivity was available, at which point all data automatically transferred and aggregated to cloud servers and portal for analysis and remote quality assurance.

### Data analysis

The preliminary data were analyzed using SPSS 20 (Statistical Package for the Social Sciences) [24] and Epi Info [25]. Descriptive results were presented as frequencies and percentages. Chi-square tests were used to compare the yield of disease between different subgroups. In future analyses to evaluate the triage tests, the area under the curve (AUC) on a ROC (Receiver Operating Characteristic) curve will be used.

Additional details on study methods (i.e. study site, organization of the clinics, training of staff, and image collection protocol) are provided in the Additional file 1.

## Results

A total of 9625 women came for screening, of which 9406 (97.7%) eligible women aged 30–49 years were screened; and 219 (2.3%) ineligible women (1.7% due to age restrictions) were not screened (Details on enrollment and exclusions are provided in the Additional file 1). A total of 442 (4.7%) of 9406 women living with HIV (WLWH) were noted when cross referenced with the HIV clinic of OAUTHC, whereas HIV status was unknown for the remaining 8964 (95.3%) women.

With self-sampling, we were able to screen an average of 20 women per working day (with a peak of up to 100 women in a single day per clinic with two self-sample collection areas). Roughly 9065 participants provided written feedback. Of those, 80.8% women said that they were able to collect the self-sample without any help from a nurse and 81.2% women said that they would prefer self-sampling to provider’s sampling in the future. Asked to score their impressions, 78.5% women were very confident in their ability to collect the self-sampling, 91% found it very easy, 88.5% found it very comfortable (not painful at all), 95.1% found the video to be very helpful in guiding how to collect the sample, and 97.8% said they are very likely to recommend self-sampling to others. The difficult components of self-sampling reported (one or more issues for 2322 respondents) were: the decision on how deep to insert the brush into the vagina for 52% of 2322, how to insert the brush into the vagina for 49.1%, identifying the vaginal opening for 39.6%, rotating the brush inside the vagina for 23.3%, and proper handling to put the brush into the tube after collection for 18.9% women.

A total of 1630 (17.3%) [95% confidence interval (CI):16.6–18.1%] of 9406 screened women were HC2-positive. The rate of HC2 positivity was 16.2% among the 8964 women with unknown HIV status and 40.5% among the 442 women living with HIV (*P* < 0.001, independent chi-square) (Table 1, Table 2). The strong differences in HPV positivity between the two groups persisted in all age groups.

**Table 1:** Age-specific prevalence of high-risk HPV and precancer among women with unknown HIV status

Age at screening (years)	No. of women screened in the age group	No. of HPV-positive (% of women screened) (95% CI)	Colposcopy attendance among HPV-positive	Histopathology results^**b**^ (% of women attending colposcopy^**c**^) (95% CI)		Yield of the screening effort^**b**^ (% of total women screened)	
				CIN2+	CIN3+	CIN2+	CIN3+
**30–34**	2553	476 (18.6%) (17.2–20.2%)	393 (82.6%)	26 (6.6%) (4.4–9.6%)	14 (3.6%) (2.0–5.9%)	1.0%	0.5%
**35–39**	2646	411 (15.5%) (14.2–17.0%)	354 (86.1%)	18 (5.1%) (3.0–7.9%)	10 (2.8%) (1.4–5.1%)	0.7%	0.4%
**40–44**	2282	351 (15.4%) (13.9–16.9%)	309 (88.0%)	12 (3.9%) (2.0–6.7%)	5 (1.6%) (0.5–3.7%)	0.5%	0.2%
**45–49**	1479	213 (14.4%) (12.7–16.3%)	194 (91.1%)	15 (7.7%) (4.4–12.4%)	7 (3.6%) (1.5–7.3%)	1.0%	0.5%
**Total**	8960^a^	1451 (16.2%) (15.4–17.0%)	1250 (86.1%)	71 (5.7%) (4.5–7.1%)	36 (2.9%) (2.0–4.0%)	0.8%	0.4%

^a^Not including four women with missing data on age

^b^CIN2+ and CIN3+ include a total of four cases of squamous cell carcinoma (two of them were in 49-year old women, one in a 47-year old woman, and one in a 33-year old woman; all four cases were diagnosed on biopsy). We also presumptively included five women with OncoE6 HPV 16 positivity because of the known high positive predictive value of the biomarker (Three of the five had confirmed CIN2+; in two LLETZ was recommended and is still incomplete)

^c^Histopathology report was not completed for four participants due to COVID-19 pandemic spread and lockdown. These four participants were excluded from the denominator

**Table 2:** Age-specific prevalence of high-risk HPV and precancer among women living with HIV (WLWH)

Age at screening (years)	No. of women screened in the age group	No. of HPV-positive (% of women screened) (95% CI)	Colposcopy attendance among HPV-positive	Histopathology results^**a**^ (% of women attending colposcopy^**b**^) (95% CI)		Yield of the screening effort^**b**^ (% of total women screened)	
				CIN2+	CIN3+	CIN2+	CIN3+
**30–34**	77	35 (45.5%) (34.1–57.2%)	27 (77.1%)	1 (3.8%) (0.1–19.6%)	1 (3.8%) (0.1–19.6%)	1.3%	1.3%
**35–39**	160	57 (35.6%) (28.2–43.6%)	41 (71.9%)	2 (5.0%) (0.6–16.9%)	2 (5.0%) (0.6–16.9%)	1.3%	1.3%
**40–44**	134	46 (34.3%) (26.3–43.0%)	40 (87.0%)	4 (10.0%) (2.8–23.7%)	3 (7.5%) (1.6–20.4%)	3.0%	2.2%
**45–49**	71	41 (57.7%) (45.4–69.4%)	35 (85.4%)	4 (11.4%) (3.2–26.7%)	3 (8.6%) (1.8–23.1%)	5.6%	4.2%
**Total**	442	179 (40.5%) (35.9–45.2%)	143 (79.9%)	11 (7.8%) (4.0–13.5%)	9 (6.4%) (3.0–11.8%)	2.5%	2.0%

^a^CIN2+ and CIN3+ includes one case of squamous cell carcinoma in a 49-year old woman

^b^Histopathology report was not completed for two participants due to COVID-19 pandemic spread. These two participants were excluded from the denominator

Out of 1630 HPV-positive women, 1400 (85.9%) enrolled for the study at colposcopy, of which seven cases were excluded due to unsatisfactory colposcopy and difficulty in image collection due to various reasons outlined in the Additional file 1. Out of the 1400 who enrolled, 709 (50.6%) women came for colposcopy after only one scheduling contact. An additional 299 (21.4%), 166 (11.9%), 85 (6.1%), 77 (5.5%) and 64 (4.6%) came after two, three, four, five, and more than five contact attempts.

Following the protocol of immediate management by colposcopic impression, without awaiting histopathology diagnosis of the biopsies taken, 114 (8.2%) women were treated with thermal ablation and 78 (5.6%) with LLETZ.

Overall, CIN2+ and CIN3+ (including five cancers), were detected in 5.9% (95% CI:4.7–7.3%) and 3.2% (95% CI:2.4–4.3%) of the HPV-positive women attending colposcopy (85% attendance among the 1630), respectively. Thus, overall yield of the screening effort was 0.9% of 9406 women for CIN2+ and 0.5% for CIN3 + .

The prevalences of CIN2+ and CIN3+ among the HPV-positive women with unknown HIV status attending colposcopy were 5.7 and 2.9%, respectively, whereas the overall yields of the screening effort in these groups were 0.8 and 0.4%, respectively (Table 1). The age-specific prevalences of high-risk HPV positivity and precancer among women with unknown HIV status are shown in Table 1. The prevalence of HPV decreased from 18.6% at age 30–34 years to 14.4% at age 45–49 years (*P* = 0.0003, chi-square for trend). No meaningful trend was observed in the prevalence of precancer by age (*P* = 0.87, chi-square for trend for CIN2+), except for a very high 24.1% (seven CIN2+ including three cancers) overall prevalence at self-reported age of 49 years.

The prevalences of CIN2+ and CIN3+ among the HPV-positive WLWH attending colposcopy were 7.8 and 6.4%, respectively (*P* = 0.31 for CIN2+ and *P* = 0.03 for CIN3+, independent chi-square test in comparison to women with unknown HIV status) (Table 2). The yield of the screening effort among women living with HIV was 2.5% for CIN2+, of which all except two were actually CIN3+ (*P* < 0.001 for CIN2+ and CIN3+, independent chi-square in comparison to women with unknown HIV status) (Table 2). Relatively small age-specific numbers preclude any conclusions regarding a trend in the prevalence of HPV or precancer by age. The proportion of HPV-positive women increased with decrease in CD4 (cluster of differentiation 4) count (64% in CD4 < 200/mm^3^ vs 31.1% in CD4 > 500/ mm3, *P* = 0.001, chi-square for trend) and increase in HIV viral load (36.3% in <=20 copies/ml vs 50% in > 20 copies/ml, *P* = 0.02, independent chi-square), however small numbers precluded trend analysis of precancer/cancer.

**Fig. 1:**
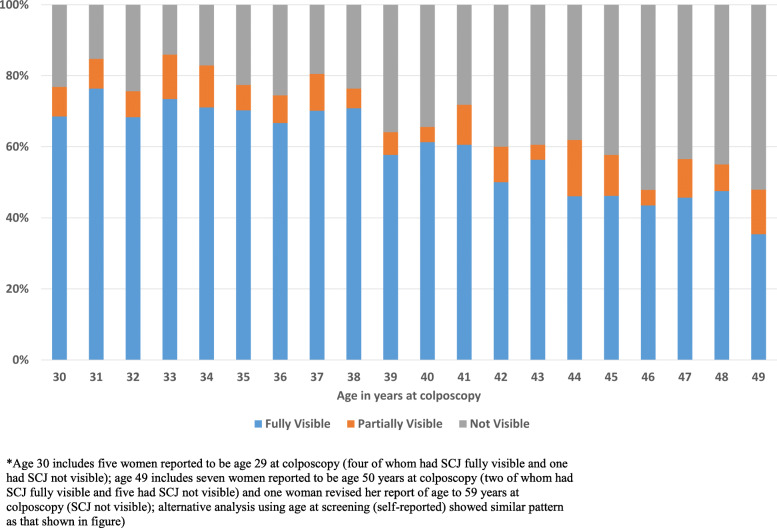
Squamocolumnar junction visibility by age (*n* = 1393)

**Fig. 2:**
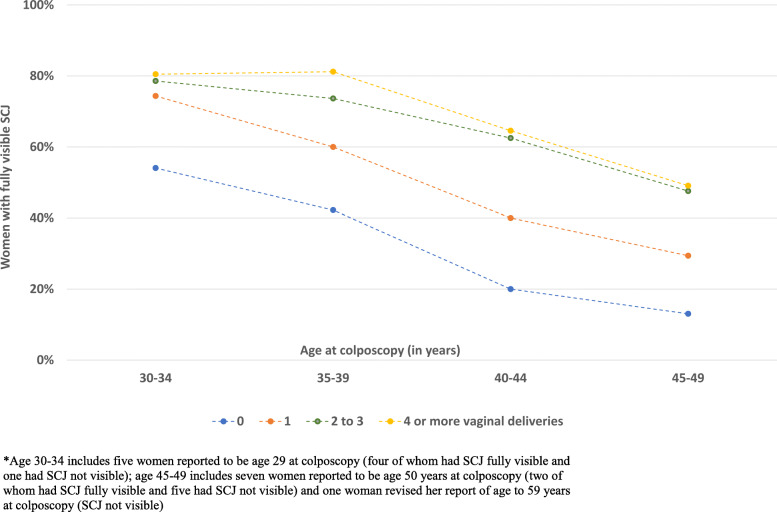
Squamocolumnar junction (SCJ) visibility, by age and parity (*n* = 1380)

Out of a total of 75 histopathologically confirmed cases, 59 (78.7%) were diagnosed through biopsy or LLETZ of acetowhite lesions at colposcopy visit, 35 of which (59.3% of 59) had a high-grade colposcopic impression. QA review of colposcopic images revealed potential under-biopsying of more subtle acetowhite lesions. 16 (21.3%) of 75 histopathologic precancer cases were identified, in the absence of visible acetowhitening, only on ECC (1.2% of colposcopy examinations and 0.2% of the screening population). This is important in light of the fact that even amongst women as young as age 30, the SCJ was only partially visible in 8.3% and not visible in 23.1% of women (31.5% total), rising to 12.5 and 52.1% (64.6% total) by age 49 years (P < 0.001, chi-square for trend) (Fig. 1). Interestingly, multiple vaginal deliveries were found to increase full SCJ visibility (Fig. 2).

Among 192 women treated on-site based on colposcopic impression, without awaiting histopathology results, only 48 (25%) were eventually diagnosed with CIN2+ on histopathology (Table 3). Viewing overtreatment from another perspective, 11% of women with histopathologic <CIN2 were treated based on colposcopic impression. On the other hand, 43.2% of CIN2 (16 out of 37) and 35% of CIN3 (14 out of 40) were not treated immediately; either because of underdiagnosis [diagnosed later on ECC] or a variety of programmatic issues such as equipment failure. A total of 12 (1.1% of colposcopy population) precancer cases needed more than one treatment visit in order to obtain clear margins free of high-grade findings.

**Table 3:** Management of women by histopathologic diagnosis

Histopathologic diagnosis		Management					Row total
		No treatment indicated (row%) (column%)	Immediate management based on colposcopic impression (row%) (column%)		Delayed excisional treatment on a recall visit (row%) (column%)		
			With ablation	With LLETZ	Completed	Pending^**a**^	
**<CIN2**		1158 (88.7%) (100.0%)	90 (6.9%) (78.9%)	54 (4.1%) (69.2%)	3^c^ (0.2%) (12.0%)	0	1305 (100%)
**CIN2**	**Diagnosed on biopsy/LLETZ**	0	10 (40.0%) (8.8%)	11 (44.0%) (14.1%)	2 (8.0%) (8.0%)	2 (8.0%) (16.7%)	25 (100%)
	**Diagnosed on ECC**	0	0	0	8 (66.7%) (32.0%)	4 (33.3%) (33.3%)	12 (100%)
**CIN3**	**Diagnosed on biopsy/LLETZ**	0	13 (38.2%) (11.4%)	12 (35.3%) (15.4%)	5 (14.7%) (20.0%)	4 (11.8%) (33.3%)	34 (100%)
	**Diagnosed on ECC**^**b**^	0	1 (16.7%) (0.9%)	0	3 (50.0%) (12.0%)	2 (33.3%) (16.7%)	6 (100%)
**Cancer**	**Diagnosed on biopsy/LLETZ**	0	0	1^d^ (20.0%) (1.3%)	4 (80.0%) (16.0%)	0	5 (100%)
**Column total**		1158 (100%)	114 (100%)	78 (100%)	25 (100%)	12 (100%)	**Grand total = 1387**

^a^Recall attempts are temporarily paused due to COVID-19 pandemic spread

^b^Includes two women with OncoE6 HPV 16 positive, in whom LLETZ was recommended on recall and is still incomplete

^c^Includes one case with colposcopic impression of cancer treated with hysterectomy of what eventually turned out to be <CIN2; other two cases were recalled for LLETZ due to reporting error

^d^Invasive squamous cell carcinoma with CIN3 at margins was diagnosed on on-site LLETZ leading to a recall for a repeat LLETZ with CIN3 diagnosis leading to a 2nd recall for a hysterectomy

Out of 1138 (82%) patients with full clinical quality assurance review completed, 143 (12.6%) cases were recommended for recall; mainly (113, or 9.9% of total) for repeat ECC; commonly because the tissue was insufficient for diagnosis on the prior ECC (100, or 8.8% of total) or because the ECC required dilatation/sedation due to stenotic os or tethered cervix (13, or 1.1% of total). However, the overall yield of precancer from recall for ECC was only 0.2% for the colposcopy population and 0.02% for the general population of women aged 30–49 years.

## Discussion

Our results showed the feasibility and acceptability of self-sampled HPV testing and smartphone-based cervical image collection in Nigeria. Nevertheless, the combination of higher HPV prevalence (17.4%) with a much lower risk of precancer (0.9%) suggests the need for triage to improve the accuracy of the screening program. In addition, any visual triage method would require restricting the upper age limit of inclusion to increase program effectiveness.

There was a very positive response and clear acceptability for vaginal self-sampling for HPV testing. The difficult aspects of self-sampling reported by some participants included identifying the vaginal opening, determining depth of the insertion of the brush into the vagina, rotating the brush inside the vagina, and putting the brush back into the tube after collection; these need to be explained more clearly in future self-sampling programs. Compliance with follow-up for colposcopy was also very high.

The two previous phases of Project Itoju established the epidemiology of HPV infection in rural Nigeria [26], validated a low-cost HPV test (careHPV, Qiagen) [27] (phase 1) and attempted (ultimately unsuccessfully) to determine whether immunosuppression due to soil-borne helminth infections or other parasitoses was the cause of high HPV prevalence among older women in the region (phase 2). In the current phase of Project Itoju, we reconfirmed the overall high prevalence of HPV infections even after restricting the age of screening to 30–49 years of age [28]. Although the prevalence of HPV decreased to 16% at age 45–49 years from 19% at age 30–34 years, it was still much higher than the global average of around 5% for women age 45–54 years and consistent with what is observed for the women in sub-Saharan Africa [29]. Despite the high prevalence of HPV infection, the prevalence of precancer was less than 1% in the general population of mostly unscreened women 30–49 years old. One reason for the low prevalence of precancer could be under biopsy of more subtle acetowhite lesions [30]. It also underscores the need to further study the type-specific natural history in the region of HPV acquisition, clearance, persistence, and progression, particularly in the setting of HIV infection. In this current study, we obtained samples for HPV genotyping from women at screening and again at colposcopy visit. We will be testing and reporting the results of these tests elaborating the prevalence of various HPV genotypes and short-term clearance of infection in subsequent papers. We also hope to retest the women in the future to explore the long-term persistence of HPV infection.

The prevalence of HPV and precancer (particularly CIN3+) was markedly high among WLWH as also reported by others [28]. This was observed despite the suppressed viral load with a documented average of < 20 copies/ml. This finding was expected since HPV and HIV can be co-transmitted and women with HIV tend to have lower clearance of acquired HPV infections. The management of HPV in WLWH is an important topic beyond the scope of this article.

**Fig. 3:**
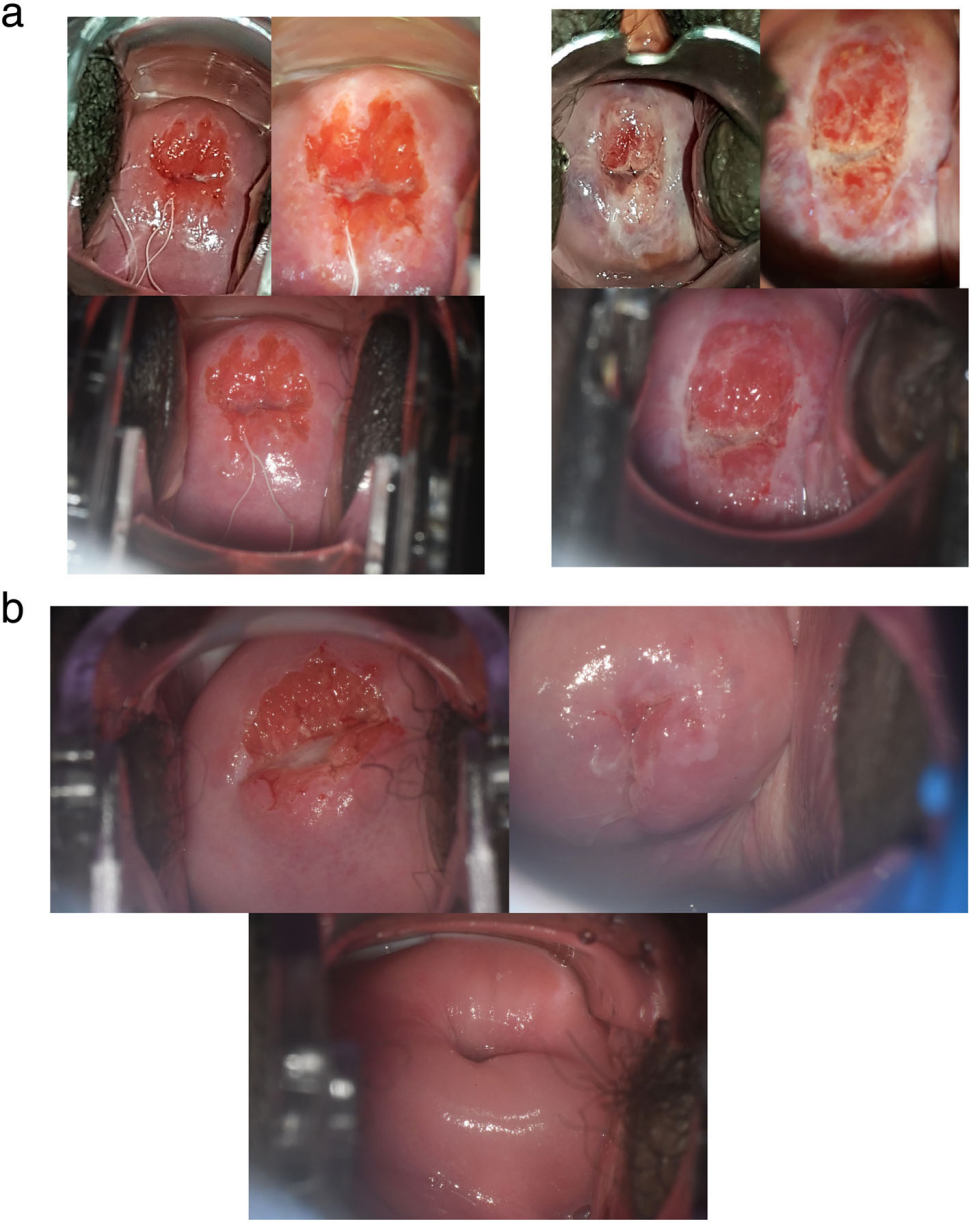
Effect of image capture method on cervical appearance and limitation of visual triage method. **a**. Cervical images of the same cervix showing histopathologic <CIN2 (left trio) and CIN2+ (right trio), captured with (clockwise starting from upper left) a Samsung S8 smartphone camera and its flashlight, a MobileODT EVA device (Samsung J5 phone with an extra light source and a zoom lens), and a Zeiss FC150 colposcope with a beam splitter and DSLR camera (see supplement). **b**. Cervical images of histopathologic CIN2+ cases with squamocolumnar junction (clockwise from upper left) fully visible, partially visible, and not visible (diagnosed on ECC); captured with a Zeiss FC150 colposcope with a beam splitter and DSLR camera

According to the World Health Organization (WHO), Nigeria has only four physicians per 10,000 population [31]. At present, only < 10% of women > 15 years have ever been screened for cervical cancer in Nigeria [32]. In a resource-constrained setting with a shortage of expert gynecologic providers and infrastructure, avoidable referral to colposcopy and substantial overtreatment are not sustainable. On the contrary, in settings with once in a lifetime screening opportunity for a majority of women, substantial missed diagnosis and undertreatment is not acceptable either. It is therefore essential that simple yet accurate triage tests, separately or in combination, are available to stratify risk of precancer/cancer among HPV-positive women such that treatment intensity can be tailored to risk of cancer and sustained with local resources. In the future, we will be assessing the machine-learning based AVE of cervical images using three different image collection methods. We will be reporting the results of the assessment of the combination of AVE with HPV genotyping for triage of HPV-positive women in subsequent papers. However, preliminary analysis of the cervical images from three different devices (Fig. 3a) has raised an important research question regarding the device portability of deep-learning based AVE algorithms due to variation in color, brightness, reflection, glare imparted by each device camera, light source and image processing application. Training or automation to capture an in-focus, non-blurry good quality cervical image, capturing entire SCJ, for evaluation by AVE, is yet another challenge.

It is important to note that AVE, like other visual assessment methods, requires that the cervical SCJ be visible. In the current study population, we found that the SCJ was not fully visible for almost 64.6% of women by age 49 years. We also corroborated an earlier poorly understood observation that women with multiple vaginal deliveries were more likely to have fully visible SCJs [33]. No visual screening methods or ablative treatment methods work when the transformation zone, where cervical cancer typically arises, is not visible (Fig. 3b) [12, 34]. This could partially explain the decrease in the prevalence of precancer with age despite the high HPV prevalence, found in this study. It is, therefore, particularly crucial to emphasize restricting visual approaches for screening or triage of older women to avoid giving false reassurance to women in these age groups and to avoid identifying high-risk HPV-positive women, particularly with HPV 16, 18/45, with no available means of further triage. It is worth noting however that even at age 30, the SCJ was not fully visible in up to 30% of women, thus age-restriction to women <=49 years does not eliminate inadequacies in visual triage. The current standard practice is to collect an ECC sample whenever the SCJ is not fully visible, to rule out cancer within the endocervical canal. But an ECC needs an interpretation by an expert histopathologist, which is challenging in low resource settings. Difficulty in collecting ECC in women with a stenotic cervical os and insufficient tissue in the ECC sample to rule out carcinoma are other challenges with ECC. Thus, the development of low-cost simple triage alternatives for ECC remains an unsolved challenge for improving cervical cancer screening programs. Research is also needed on simpler alternatives for LLETZ, that could be performed easily by a general physician or a nurse without the need of an expert gynecologist, particularly in low-resource settings where the needed resources are in short supply especially in rural underserved areas.

It is worth noting that even though the yield of precancer from ECC at colposcopy visit was 3.2% (21% of total precancers diagnosed on ECC, possibly due to not taking enough biopsies of subtle acetowhite lesions), the additional yield of precancer from the women recalled for insufficient ECC or difficult ECC collection was only 0.02% for the overall screened population. Another study has also noted overall low yield of ECC with an increase in proportionate additional yield of ECC when fewer biopsies are taken [35]. It is worth exploring the cost-effectiveness of recalls for ECC to avert a very low risk of adenocarcinoma in low-resource settings where no organized follow-up is available. Any recommendation for follow-up outside the study in these settings is likely to remain a theoretical reassurance. In this regard, there were a total of 12 women (1.1% of colposcopy population) who needed multiple rounds of treatment in order to obtain clear margins on excision. These are women with confirmed high-grade lesions and hence also at high risk of progression to invasion. Even though in the study we managed to achieve a relatively higher rate of compliance with follow-up, the compliance in the real-world setting needs to be monitored and factored in the effectiveness of the screening programs.

We had to halt the recall activities under the screening program as the global spread of the COVID-19 pandemic led to pausing of field efforts in Nigeria, including a very few treatment visits still pending, and changed the risk-benefit ratio for cervical cancer screening. When we reopen, the first step will be to complete these treatment visits. Then, moving forward in the COVID-19 era, we are considering the importance of self-sampled HPV testing with a sterile kit at a household level, avoiding any mass gathering and minimizing the need for speculum examination. A small percentage of HPV-positive women with high-risk types could be triaged and treated, spacing community clinic appointments in a COVID conscious manner [36].

The limitations of this study should be noted. The population selected for the study was a volunteer population of women residing in the university town of Ile-Ife, which may have a lower HPV prevalence than the general population in Nigeria. Also, HC2 is not generally used for self-sampling, as it is slightly less sensitive for detection of precancer than PCR-based HPV test methods [37]. A moderate amount of cross-reactivity against other genetically related but less oncogenic HPV types with HC2 is well-documented. This may partly explain the low precancer to HPV ratio found in this study. Despite the known limitations of HC2, its operational simplicity, and easy trainability, as supported by virtually trouble-free operation of HC2 throughout the study supported its use.

In future work we will assess misclassification by retesting residual screening samples with a more sensitive whole-genome sequencing method [20]; review and recall women with highest risk HPV types and potential missed biopsies of subtle acetowhite lesions; and digitalize the histopathology slides for a second review, particularly for borderline cases.

## Conclusion

A cervical cancer screening program using self-sampled HPV testing, with colposcopic immediate management of women positive for HPV, is feasible in Nigeria but results in both over-and under-treatment. There are newer HPV tests entering the marketplace that cost less than ten dollars per test, take less than an hour to perform, and provide genotyping, suggesting that a self-sampled HPV based screening program would be feasible in this population in the future [38]. Having proven feasibility, we are now evaluating the accuracy and efficacy in diagnosing CIN2+ of smartphone-based automated visual evaluation of cervical images combined with HPV genotyping as an assistive strategy to improve visual triage.

## Supplementary information

**Additional file 1.**

## Data Availability

The datasets used and/or analysed during the current study are available from the corresponding author on reasonable request.

## Abbreviations

AUC: Area under the curve
AVE: Automated visual evaluation
CD4: Cluster of differentiation 4
CI: Confidence interval
CIN: Cervical intraepithelial neoplasia
COVID-19: Coronavirus disease of 2019
DNA: Deoxyribose nucleic acid
DSLR: Digital single lens reflex
ECC: Endocervical curettage
EVA: Enhanced visual assessment
HC2: Hybrid capture-2
HIPAA: Health Insurance portability & accountability act
HPV: Human papillomavirus
HIV: Human immunodeficiency virus
LLETZ: Large loop excision of the transformation zone
NCI: National Cancer Institute
OAUTHC: Obafemi Awolowo University Teaching Hospitals Complex
OAU: Obafemi Awolowo University
PCR: Polymerase chain reaction
RLU/CO: Relative light unit/cut-off
RNJMS: Rutgers New Jersey Medical school
ROC: Receiver operating characteristic
SCJ: Squamocolumnar junction
SPSS: Statistical package for social studies
STM: Specimen transport media
US: United States
WLWH: Women living with HIV
