# Supplementary material for: Design and feasibility of a novel program of cervical screening in Nigeria: self-sampled HPV testing paired with visual triage
Source: Infect Agent Cancer. 2020 Oct 14;15:60. doi: 10.1186/s13027-020-00324-5 (PMC7556552; doi:10.1186/s13027-020-00324-5)
Supplement: Supplementary file 1 — Additional file 1. [file 13027_2020_324_MOESM1_ESM.docx]

**Additional File 1**

***Materials & Methods (Additional details):***

Study site

Ile-Ife is a large university town, approximately 218 kilometers Northeast of Lagos (1). The study colposcopy clinic was located at the gynecology unit of the Obafemi Awolowo University Teaching Hospitals Complex (OAUTHC), which is a leading multi-specialty teaching referral hospital in the region (2). The three study screening clinics were located at the OAUTHC and Obafemi Awolowo University (OAU) campus health center in Ile-Ife and the Wesley Guild Hospital in a nearby town of Ilesa, approximately 43 km Northeast from Ile-Ife.

Organization of the clinics and laboratory

Each screening clinic was staffed with a nurse and two research assistants, all of whom were female to increase the comfort level of the screened women. Each screening clinic had a self-sample collection area (a clean washroom or an examination area created with blinds to ensure privacy) equipped with a handwashing facility, a stool to place leg while self-sampling, test-tube racks, alcohol wipes to clean racks, and pictorial displays on how to self-sample. In addition, each screening clinic had a waiting and registration area with a monitor displaying a video on how to self-sample. Banners and signages were placed outside the screening clinics to direct the visitors to the clinics.

The colposcopy clinic was staffed with two study gynecologists, an enrollment nurse, two clinical nurses, two communication nurses, a project manager, and a cleaner. The colposcopy clinic examination room was equipped with a colposcopy examination table, a Zeiss FC150 colposcope (3) connected via a beam splitter to Nikon D750 digital single-lens reflex (DSLR) camera (4), a ‘case air wireless tethering system’ (5) to transfer the images from DSLR camera to the data collection application on a cellphone, an enhanced visual assessment (EVA) system with a tripod (6), a Samsung S8 phone (7) with a case (8) mounted on a tripod (9) , two Large Loop Electrosurgical generators including one battery-operated portable device (10), a thermoablation and a cryopen device, and other essential colposcopy instruments and consumables. At the beginning of each day, the nurse in the colposcopy examination room followed and completed a checklist to set up the devices for image collection.

The HPV laboratory was staffed with two laboratory technicians and equipped with a Qiagen Hybrid Capture-2 (HC2) platform, a water bath, a microcentrifuge, three 2-8^0^c refrigerators, and an air-conditioner. A solar power backup was arranged for the laboratory and the colposcopy clinic.

Training of the study staff

A detailed field procedures manual and technical guide videos were developed. All the study staff were trained on the field protocol and use of the data management system by mock practice rounds. The lab technicians were trained on HC2 HPV testing by Qiagen. Colposcopy experts from RNJMS (Rutgers New Jersey Medical school) worked with the study gynecologists for quality assurance of colposcopy examination and treatment protocols. Experts from MobileODT (12) and Global Good worked with the study gynecologists to optimize the quality of collected images. A series of placards outlining workflows and daily checklists was prepared and displayed in the work area for quick reference.

Cervical image collection protocol

Cervical images were collected with three different devices one minute after applying 5% acetic acid before each device as follows (Supplementary Figure 1): i) Samsung Galaxy S8 (7) phone mounted on a tripod, using open camera application (11) and the light of the phone camera. The in-focus images were collected using the digital zoom of the camera app, and movement of the tripod handles to adjust the distance of cellphone from the cervix. The open camera app collected three snaps of the images after one click, to avoid the motion blur effect on the images; ii) MobileODT EVA colposcope (6) mounted on the stand with its in-built light source. The in-focus images were collected using both the digital and optical zoom lens of the device. Motion blur was avoided by using the hand-wave feature of the device to collect the images; iii) Nikon DSLR D750 camera (4) attached with the Zeiss FC150 colposcope (3) via a f340 SLR-adapter with beam splitter. The in-focus images were collected using the optical zoom of the colposcope. The Case Air wi-fi tethering system application (5) on the cellphone was used to take the pictures to avoid the motion blur and to transfer the images from the D750 camera to the EVA app on a cellphone. The EVA app used for data collection had a timer on the camera screen. The user could focus the camera for image collection while waiting for one minute at which point the phone vibrated to signal image collection.

Study enrollment and exclusions

Details on number of women enrolled for screening and colposcopy, ineligibility due to various reasons, and refusal for consent are provided in supplementary figure 2.

**Supplementary Figure 1: Colposcopy room workflow**


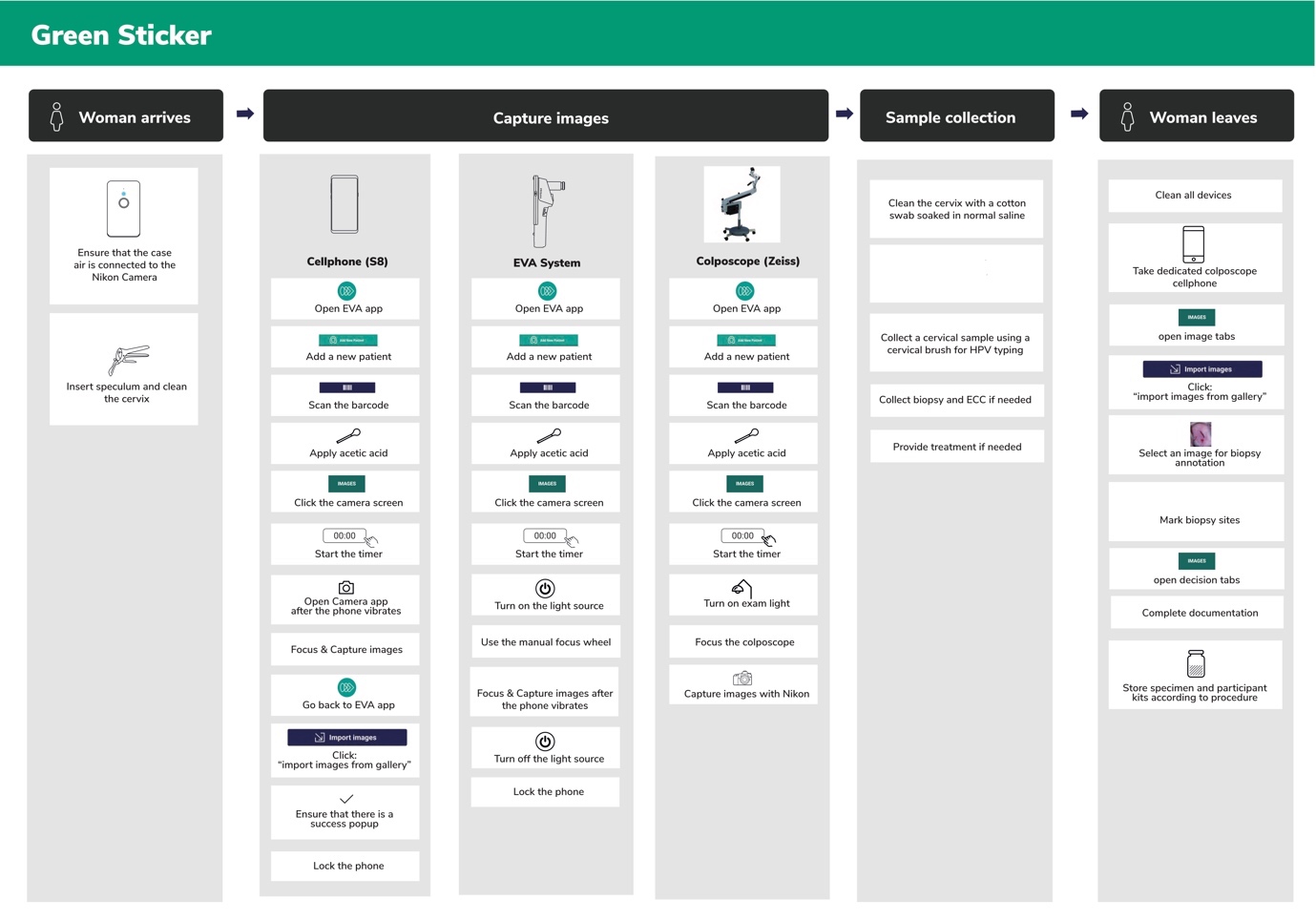


**Supplementary Figure 2: Enrollment and exclusion at various stages in screening and colposcopy**


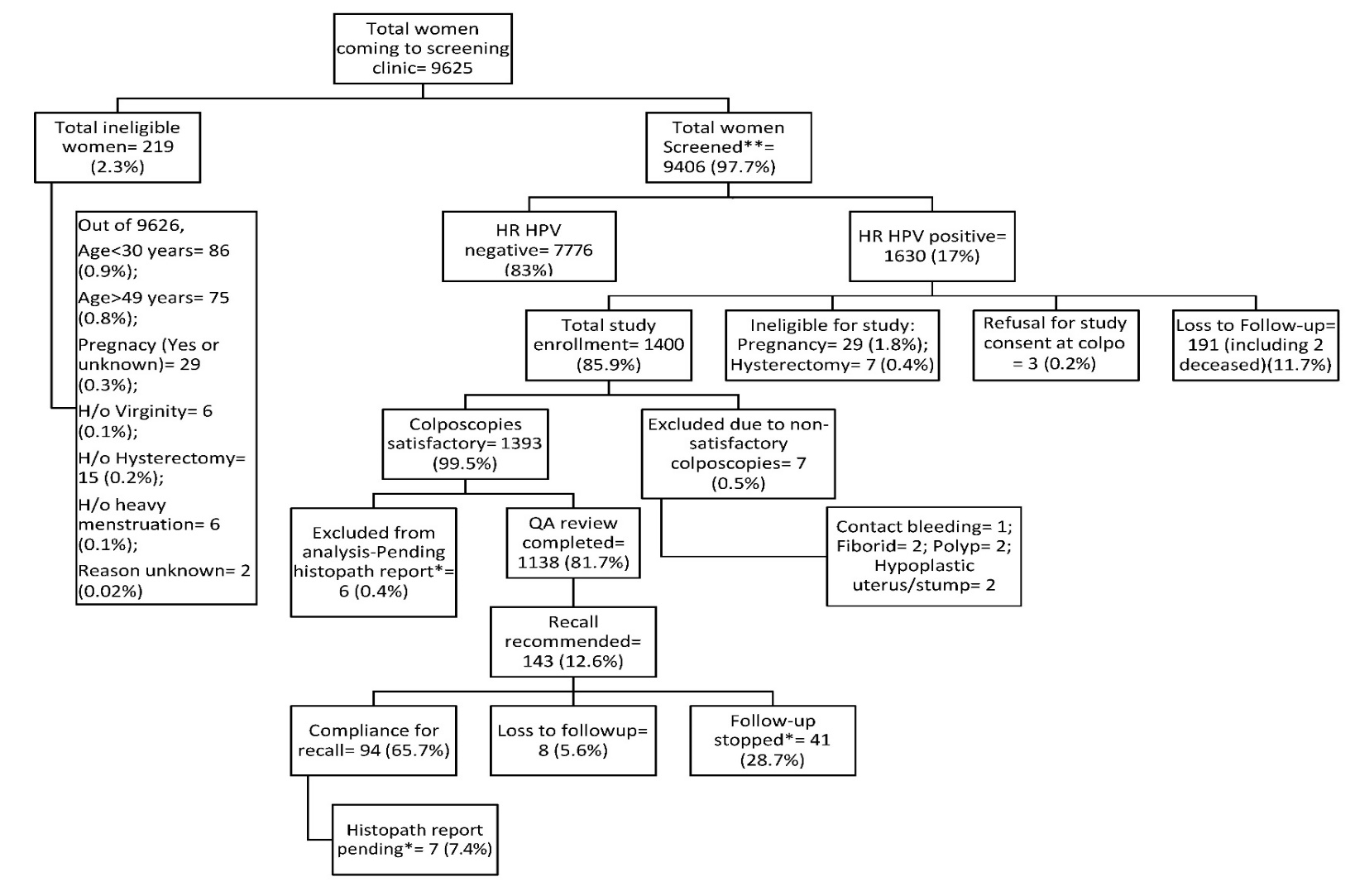


*study activities paused due to COVID-19 pandemic spread and lockdown**40 (0.4%) (all except one were HPV negative) of 9406 women refused the additional consent obtained at screening to store residual sample after HC2 test and 15 (0.2%) (all HPV negative) of 9406 women refused the additional consent obtained at screening to be contacted in future for a follow-up study

**References:**

1. Ile-Ife | Nigeria | Britannica [Internet]. [cited 2020 Mar 17]. Available from: https://www.britannica.com/place/Ile-Ife

2. Welcome to OAUTHC [Internet]. [cited 2020 Mar 17]. Available from: http://oauthc.com/readmore.aspx

3. Colposkop 150 FC [Internet]. Carl Zeiss Meditec, Inc. [cited 2018 Mar 12]. Available from: https://www.zeiss.com/meditec/us/products/gynecology-/colposcopy/colposkop-150-fc.html#technical-data

4. Nikon Inc. Nikon D700. Nikon Inc. 2018.

5. Tether tools. Case Air Wireless Tethering System. Tether Tools, Inc. 2018.

6. EVA COLPO | MobileODT [Internet]. [cited 2020 Mar 26]. Available from: https://www.mobileodt.com/products/eva-colpo/

7. Gadgets 360. Samsung Galaxy S8 [Internet]. Gadgets 360-An NDTV Venture. [cited 2018 Mar 2]. Available from: https://gadgets.ndtv.com/samsung-galaxy-s8-4009

8. mophie External Battery Case for Samsung Galaxy S8 [Internet]. Best Buy. 2018 [cited 2018 Mar 9]. Available from: https://www.bestbuy.com/site/mophie-external-battery-case-for-samsung-galaxy-s8-black/6144417.p?skuId=6144417

9. Manfrotto 65" Compact Advanced Tripod [Internet]. Best Buy. 2018 [cited 2018 Mar 9]. Available from: https://www.bestbuy.com/site/manfrotto-65-compact-advanced-tripod-black/4853012.p?skuId=4853012

10. CureMedical. New-ESU-110-Brochure.pdf [Internet]. [cited 2020 Apr 10]. Available from: https://docs.google.com/viewerng/viewer?url=http://www.curemedicalglobal.com/wp-content/uploads/2019/11/New-ESU-110-Brochure.pdf&hl=en

11. Open Camera [Internet]. [cited 2020 Mar 26]. Available from: https://opencamera.org.uk/
